# Evaluating digestibility and toxicity of native warm-season grasses for equines

**DOI:** 10.1093/tas/txaa224

**Published:** 2020-12-03

**Authors:** S M Ghajar, H McKenzie, J Fike, B McIntosh, B F Tracy

**Affiliations:** 1 School of Plant and Environmental Sciences, Virginia Polytechnic Institute & State University, Blacksburg, VA; 2 Department of Large Animal Clinical Sciences, Virginia Polytechnic Institute & State University, Blacksburg, VA; 3 Mars Equestrian™, McLean, VA

**Keywords:** equine, forage, native grasses, pasture

## Abstract

Introduced cool-season grasses are dominant in Virginia’s grasslands, but their high digestible energy and nonstructural carbohydrate (NSC) levels pose a risk for horses prone to obesity and laminitis. Native warm-season grasses (NWSGs) have lower digestible energy and NSC levels that may be more suitable for horses susceptible to laminitis. Although NWSGs have desirable characteristics, they are novel forages for horses. Little is known about NWSG intake or potential toxicity to horses or how grazing by horses may affect NWSG swards. The overall objectives of this research were to 1) assess voluntary intake, toxicological response, and apparent digestibility of NWSG hays fed to horses; and 2) evaluate the characteristics of three NWSG species under equine grazing. For the first objective, a hay feeding trial using indiangrass (IG) (*Sorghastrum nutans*) and big bluestem (BB) (*Andropogon gerardii*) was conducted with nine Thoroughbred geldings in a replicated 3 × 3 Latin square design. Voluntary dry matter intake of IG and BB hays by horses were 1.3% and 1.1% of BW/d, lower than orchardgrass (*Dactylis glomerata*), an introduced cool-season grass, at 1.7% of BW/d (*P* = 0.0020). Biomarkers for hepatotoxicity remained within acceptable ranges for all treatments. Apparent dry matter digestibility (DMD) did not differ among hays, ranging from 39% to 43%. NSC levels ranged from 4.4% to 5.4%, below maximum recommended concentrations for horses susceptible to laminitis. For the second objective, a grazing trial was conducted comparing IG, BB, and eastern gamagrass (EG) (*Tripsacum dactyloides*) yields, forage losses, changes in vegetative composition, and effects on equine bodyweight. Nine, 0.1-ha plots were seeded with one of the three native grass treatments, and each plot was grazed by one Thoroughbred gelding in two grazing bouts, one in July and another in September 2019. IG had the greatest available forage, at 4,340 kg/ha, compared with 3,590 kg/ha from BB (*P* < 0.0001). EG plots established poorly, and had only 650 kg/ha available forage during the experiment. Grazing reduced standing cover of native grasses in IG and BB treatments by about 30%. Horses lost 0.5–1.5 kg BW/d on all treatments. Findings suggest IG and BB merit further consideration as forages for horses susceptible to obesity and pasture-associated laminitis.

## INTRODUCTION

More than half of horses in Virginia are overweight or obese (Thatcher et al., 2008). Obesity puts a horse at risk for serious health issues such as insulin resistance and laminitis (Geor, 2009). Laminitis is a disease characterized by an inflammatory response damaging the lamellar layer of the horse’s hooves, allowing the coffin bone to rotate (Geor, 2010). It is painful, costly to treat, and may necessitate humane euthanasia of the horse.

Although the mechanisms that precipitate laminitis are not fully understood, this inflammation in the hoof usually follows the consumption of large quantities of readily-fermented carbohydrates (Geor, 2010). When a horse with a predisposition to obesity or insulin resistance consumes a large quantity of nonstructural carbohydrates (NSC)—carbohydrates not forming the walls and membranes of the plant, but rather starch and water-soluble carbohydrates—it may prove more than the intestine can readily digest, and the bolus of carbohydrates can end up being fermented rapidly in the hindgut (McIntosh, 2006). This rapid fermentation lowers hindgut pH, altering the microbiome, and causing the release of endotoxins into the bloodstream (Geor, 2010). For horses susceptible to obesity and laminitis, it is recommended not to exceed 10–12% NSC concentration in the diet (Geor, 2010). Cool-season grass pastures can easily exceed this limit throughout much of the year (McIntosh, 2006), and even grass hays commonly fed to horses such as orchardgrass (*Dactylis glomerata*) can have NSC concentrations above 12% (Martinson et al., 2012). As a result of the high NSC levels in many common forage species, pasture-associated laminitis may account for nearly half of all cases in the United States (Geor, 2009).

Warm-season grasses, which are grasses with higher optimal growth temperatures and often a C4 photosynthetic pathway, generally have lower levels of NSC than cool-season forages, and no fructan, a simple carbohydrate thought to play a role in laminitis (Kagan et al., 2011). Staniar et al. (2010) found teff hay (*Eragrostis tef*) presented adequate nutrition and low carbohydrate levels for horses. Kagan et al (2011) measured carbohydrate levels of bermudagrass (*Cynodon dactylon*) at different stages of maturity, and at different times of day, and found it to have suitably low carbohydrate levels to be considered safe for horses prone to laminitis. While these studies on introduced forage species have provided alternatives to high-carbohydrate cool-season forages for horses, no studies have examined the use of native warm-season grasses (NWSG) in equine forage systems.

NWSG were once abundant in the Piedmont of Virginia as a result of Native American use of prescribed fire to promote savannahs with abundant game species (Tompkins et al., 2010). Common species native to much of the eastern United States include big bluestem (BB) (*Andropogon gerardii*), indiangrass (IG) (*Sorghastrum nutans*), and switchgrass (*Panicum virgatum*). Though research is lacking on the use of BB or IG as equine forages, one study examining NSC in forages under different light conditions found that NSC levels in BB never exceeded 12%, suggesting some NWSG may have ideal NSC levels for horses susceptible to laminitis (Kephart and Buxton, 1996).

Though the lower NSC concentration in NWSGs may be optimal for horses prone to metabolic disorders, questions remain regarding the safety of these grasses for horses. A number of studies have determined that the Panicum genus causes hepatotoxicity in horses. Several species of grass in that genus are common and widespread throughout the eastern United States, including switchgrass, fall panicum (*Panicum dichotomiflorum*), and many species commonly referred to as “panic grasses” due to their large panicle seedheads. After 14 horses at a boarding facility in Virginia fell severely ill in 2004, the cause was determined to be their hay, which was largely comprised of fall panicum (Johnson et al., 2006). A subsequent feeding trial of fall panicum to two research horses for 12 d resulted in highly elevated biomarkers for hepatotoxicity in blood samples taken from the horses, and histology revealed bile duct hyperplasia and hepatocyte swelling (Johnson et al., 2006).

Other research found that switchgrass ingestion by horses demonstrated hepatotoxicity as well, and determined diosgenin, a steroidal sapogenin, to be the primary toxin (Lee et al., 2001). While the sapogenin is believed to be metabolized in a form that crystallizes in the liver of sheep affected by Panicum toxicity, the mechanism by which the chemical damages the equine liver is not established, though it is thought to involve apoptosis rather than crystallization (Johnson et al., 2006).

Aside from the documented toxicity of the *Panicum* genus for horses, we could find no studies linking NWSG to toxicity in horses. However, one of the common native grasses mentioned earlier, IG, is related to the *Sorghum* genus. *Sorghum* species, whether annual or perennial, have been linked to cystitis ataxia in horses, a condition wherein hydrocyanic acid causes degeneration of the nervous system (Morgan et al., 1990). This results in loss of bladder control and hind leg coordination, and is irreversible and often fatal (Adams et al., 1969). The primary chemical that is hydrolyzed into hydrocyanic acid is dhurrin, which is also found in IG seedlings, though in IG the concentration of dhurrin declines as the plant matures (Gorz et al., 1979). Nevertheless, no cases of toxicity from IG have been documented in horses, and even-aged sorghum hay has been found to be safe for horses, as the toxic compound does not survive prolonged storage (Adams et al., 1969).

In addition to the questions of safety and nutritional value of NWSGs for horses, we found no prior research on these grasses examining their response to grazing. As equine grazing can have different impacts on a pasture than cattle grazing (Bott et al., 2013), it is necessary to conduct grazing trials with horses rather than drawing conclusions from research on cattle grazing and NWSGs to understand the potential impacts of horse grazing on NWSG swards.

This research evaluated IG and BB for use as equine forages. Specific objectives were to:

1. Determine if feeding IG and BB hay causes hepatic insult to horses.
2. Determine the voluntary intake of horses fed BB and IG hay as compared to a common hay species, orchardgrass.
3. Compare the nutritive value and apparent digestibility of these NWSG species to a common cool-season grass species, orchardgrass, when fed as hay.
4. Compare forage productivity, nutritive value, and short-term trampling effects in eastern gamagrass (EG), BB, and IG swards grazed by horses, and whether bodyweight gain differed among forage types.

## MATERIALS AND METHODS

Two experiments were conducted at the Virginia Tech Middleburg Agricultural Research & Extension Center in Middleburg, Virginia to evaluate NWSG for use as hay and pasture species for horses. Protocols for both experiments were approved by Virginia Tech’s Institutional Animal Care and Use Committee.

### Experiment I—Hay Feeding Trial

A hay feeding trial was conducted in November and December of 2018 and January of 2019. A replicated Latin square design with three treatments, three periods, and nine horses was used. The nine horses were Thoroughbred geldings (9–13 yr) and 569 ± 38 kg BW. Horses were divided into high, medium, and low relative BW groups, and then one horse from each group was randomly assigned to each of the three squares such that each square had a similar mean BW.

Three hay types were used in the feeding trials. NWSG hays were IG and BB donated in July 2018 by Ernst Conservation Seeds (Meadville, PA) from pure stands normally used for seed production. A common cool-season grass hay (orchardgrass cv ‘HLR’, Barenbrug) was used for comparison. Orchardgrass hay was produced on site in May 2018 at Virginia Tech’s Middleburg Agricultural Research & Extension Center.

The study consisted of a 10-d acclimation phase and a 4-d digestibility trial. During the first 8 d of the acclimation phase (day 1 to day 8), horses were housed by a treatment group in three adjacent dry lots with access to run-in sheds for shelter, ad libitum white salt and water, and were fed their treatment hay ad libitum from round bales. On day 9, horses were moved to individual stalls (3.5 m × 3.5 m). for the remainder of the period where they were fed their treatment hay and again had ad libitum access to clean water and white salt. On day 10, horses were fitted with fecal collection harnesses (Equisan Ltd, Australia) to ensure comfort and familiarity with the harness. The harness was also designed to collect urine; however urinary analyses were not conducted in this trial. From day 9 onwards, horses had group access to a dry lot for an hour per day for exercise and social time. The digestibility trial began on day 11 and concluded on day 14. On day 15, horses were turned out together into a mixed cool-season pasture for a 2-wk washout between experimental periods. Horses were weighed on a livestock platform scale on day 1, day 8, and day 15 of each period of the experiment.

Blood samples were collected three times per period per horse—once on day 1 (baseline), day 8, and day 15 between 0700 and 0900 hours each day. Horses were not fasted prior to sampling. Samples were collected via jugular venipuncture into 10-mL vacutainer tubes, placed on ice, and driven directly to Virginia Tech’s Marion DuPont Scott Equine Medical Center in Leesburg, VA, for analysis. Plasma was analyzed for nine different markers of toxicity. The markers were selected based on past studies of *Panicum* toxicity and other common pasture-associated toxicities that caused elevated marker profiles in horse serum (Table 1) (Curran et al., 1996; Johnson et al., 2006). Results were forwarded to a veterinarian the same day to confirm that they were within acceptable ranges. Horses were also monitored daily for any changes in behavior that might have indicated an adverse response to the novel hays being tested.

**Table 1.** Biomarkers assessed to detect potential hepatic insult to horses fed novel NWSG hays in the study

Albumin
Alkaline phosphatase (ALP)
Aspartate aminotransferase (AST)
Bile acid
Direct bilirubin
Gamma glutamyl transferase (GGT)
Sorbitol dehydrogenase (SDH)
Total bilirubin
Triglycerides

A digestibility trial was conducted in the last four days of each period. Bedding was removed from stalls and fecal collection harnesses were put on each horse. Each horse was offered its treatment hay at 2.5% BW dry matter based on the BW measured on day 8 of the experimental period. Hay DM concentration was determined by taking approximately 20 cored samples per round bale being fed, drying the samples at 135 ºC for 2 h, and dividing the dried weight by the original weight. Hay was split into two daily feedings at 0800 and 2000 hours, and fed using hay nets. Orts were collected and weighed twice daily at 0700 and 1900 hours. Fecal collection harnesses were emptied at least three times daily to ensure they did not become uncomfortable for the horse, at 0600, 1400, 2000, and if needed, 0000 hours. Feces were collected in tubes lined with plastic bags which were kept shut to preserve moisture, and total fecal output was weighed for each 24-h period starting at 2000 hours the day prior to 2000 hours on the day of weighing. Two, 1-kg subsamples were collected after weighing and compositing each horse’s fecal output each day, and one of these samples was dried at 55 °C until it reached a constant weight to determine dry matter. The remaining sample was placed in a −20 °C freezer for storage, and later thawed at room temperature for a day, then dried at 55 °C and sent to Equi-Analytical (Ithaca, NY) for chemical analyses. Grab samples of approximately 50 g were collected from each hay bale daily as they were fed to horses, and these samples were composited and submitted to Equi-Analytical for chemical analyses as well.

### Experiment II—Equine Grazing Trial

The grazing trial was conducted from July through September 2019. Nine, 0.1-ha plots were established in May 2018 in a randomized complete block design with three replicates of three treatments: IG, BB, or gamagrass (GG). Plots were established on Fauquier-Eubanks and Purcellville-Tankerville soil series (fine, mixed, active mesic Typic Hapludults). Slopes at the study site ranged from 7% to 15%.

Prior to the study, the site was managed as cool-season pasture with tall fescue (*Schedonorus arundinaceus*) as the dominant species. The site was sprayed with 4.7 L/ha glyphosate the third week of April 2018. Two weeks later, prescribed fire was used to prepare a clean seedbed and ensure fescue mortality. On June 1, glyphosate was applied again at 2.3 L/ha to kill a flush of weedy species following the fire. The same week, IG and BB plots were seeded using a Truax FLEX-II no-till drill at a depth of 6 mm and subsequently rolled with a water-filled roller to ensure adequate seed to soil contact. Gamagrass seeds were soaked in a 15% hydrogen peroxide (H_2_O_2_) solution for 18 h to break seed dormancy (Klein et al., 2008), then were drained, rinsed, and transferred to a Great Plains 706NT seed drill. After a 2-d delay due to inclement weather, the GG was seeded at a depth of 2 cm.

IG and BB plots were sprayed with imazapic herbicide at a rate of 0.15 L/ha the week after seeding. GG plots were not sprayed with imazapic, as imazapic causes stunting and mortality in GG. In the second week of July, GrazonNextHL (active ingredients: 2,4-D and aminopyralid) was applied to all plots at a rate of 2.3 L/ha to control broadleaf weeds (BW). In mid-August the same year, another application of 0.3 L/ha imazapic was conducted in IG and BB plots to control crabgrass (*Digitaria sanguinalis*). No further herbicide applications were applied to the plots.

The grazing trial began in July 2019. Horses were first turned out into their plots on July 10, and removed on July 24 when the majority of plots were reduced to 20- to 30-cm stubble height. Nine (*n* = 9) Thoroughbred geldings aged 10–14 years (median: 13) and weighing an average of 550 ± 31 kg were grouped by weight to reduce variation in grazing and trampling pressure among treatments. Each group was assigned to one treatment per grazing bout, and then reassigned randomly to another treatment on the following bout. Each plot was grazed by one horse for the duration of the grazing bouts. Horses were each provided a shade structure for shelter from the weather, ad libitum access to water and white salt, and daily applications of fly repellant.

Horses were then turned out into fescue-dominated pastures to allow NWSG plots to regrow until all plots had reached at least 46 cm height. On September 4, horses were placed back on the NWSG plots and removed on September 12, once some plots were estimated to have inadequate forage to meet daily dry matter requirements of the horses grazing them.

Plots were sampled for biomass and percent cover using haphazardly-placed 20-cm × 50-cm quadrats at the start of each grazing bout and shortly after the removal of horses. At the start of the first grazing bout, nutritive samples were also collected from plots by compositing biomass subsamples and sending them to a commercial laboratory for chemical analyses (Equi-Analytical, Ithaca, NY). Biomass samples (*n* = 5 per plot) were hand-clipped at ground level and separated into standing NWSG and weedy species, then dried in a forced-air oven at 55 °C. NWSG biomass at the beginning of each grazing bout was categorized as “available forage.” Trampled biomass was also harvested from within each quadrat; however, because of the difficulty in clipping fallen NWSG, these data were discarded as unreliable. The difference between standing NWSG biomass at the start and finish of each grazing bout was classified as “forage removed” from the plot. Forage removal included both forage consumed by horses and forage that was trampled into the ground and thus “removed” from the available forage pool. Percent cover was assessed visually as standing NWSG, grassy weeds (GW), and BW (*n* = 10 quadrats per plot). Weeds were defined as any species not seeded in the plots. In assessments made after the end of grazing bouts, the percent cover of newly trampled NWSG was also assessed.

Horses were weighed on a digital livestock scale immediately before being turned out into the plots, and immediately upon removal.

### Statistical Analysis

#### Experiment I.

Nutrient compositions for the three species of hay fed were compared by one-way ANOVA. If a difference was found, Tukey’s HSD was used for pairwise comparisons. Apparent digestibility was calculated by dividing the difference between average daily total nutrient intake and average daily nutrient excretion and dividing by average daily total nutrient intake. Voluntary dry matter intake (DMI) was compared using a mixed model with treatment, period, and treatment × period as fixed effects, and horse as a random effect. Biomarkers of toxicity were calculated as the overall change between values from samples taken on day 1 (baseline) and day 15. Changes in biomarkers of toxicity were analyzed using mixed models with treatment, period, and treatment × period as fixed effects, and horse as a random effect. Mixed models with treatment and period as fixed effects and horse as a random effect were used to analyze differences in apparent digestibility. Intake, BW changes, apparent digestibility, and biomarkers of toxicity are presented as least squares means.

#### Experiment II.

 Biomass data were analyzed both separately by date, and in total using repeated-measures ANOVA. Available forage and forage removed variables were analyzed with repeated measures ANOVA to determine differences among treatments.

Percent cover was analyzed separately by date with ANOVA and pairwise comparisons made with Tukey’s HSD. Cover variables analyzed include NWSG standing cover, NWSG trampled cover, GW, and BW.

Changes in equine weight for the two grazing bouts were calculated on a per day basis to account for the differing lengths of each bout in the analysis. Weight changes were compared with ANOVA. For all analyses, differences were considered significant when *P* < 0.05.

## RESULTS

### Experiment I

#### Forage nutrient value.

The three hays used in the grazing trial were evaluated for 18 variables to estimate nutritive value for horses. Digestible energy was greater in the IG hay at 1.9 Mcal/kg compared to 1.8 for BB (*P* = 0.0345) (Table 2). However, NDF was also over 70 in both NWSG hays, while it was 66.3 in the OG hay (*P* < 0.0001). Several nutrients were also greater in the OG hay than in the NWSG hays, including Ca (*P* < 0.0001), P (*P* = 0.0009), Mg (*P* < 0.0001), K (*P* < 0.0001), and Fe (*P* = 0.0069). Starch was greatest in the IG hay (*P* = 0.0049).

**Table 2.** Mean nutrient composition values (±SE) for big bluestem, indiangrass, and orchardgrass hay treatments

	Treatment^*a*^			
Variable	BB	IG	OG	*P-*value
Dry matter, %	94.5 ± 0.5	95.0 ± 0.4	93.4 ± 4.8	0.6613
DE, Mcal/kg	1.8 ± 0.1^b^	1.9 ± 0.0^a^	1.8 ± 0.1^ab^	0.0345
CP^*b*^, %	8.8 ± 1.7^b^	7.4 ± 0.9^b^	13.9 ± 1.0^a^	<0.0001
ADF^*c*^, %	49.4 ± 3.8^a^	44.6 ± 2.7^b^	41.3 ± 2.3^b^	0.0013
NDF^*d*^, %	76.1 ± 2.5^a^	74.6 ± 2.2^a^	66.3 ± 2.0^b^	<0.0001
Ca, %	0.2 ± 0.1^b^	0.3 ± 0.0^a^	0.4 ± 0.1^a^	<0.0001
P, %	0.2 ± 0.0^b^	0.2 ± 0.0^b^	0.3 ± 0.0^a^	0.0009
Mg, %	0.1 ± 0.0^b^	0.1 ± 0.0^c^	0.2 ± 0.0^a^	<0.0001
K, %	2.1 ± 0.3^b^	1.6 ± 0.3^c^	2.9 ± 0.1^a^	<0.0001
Fe, PPM	80.7 ± 27.3^b^	97.6 ± 10.4^b^	197.3 ± 90.7^a^	0.0069
Zn, PPM	21.5 ± 4.5	19.2 ± 1.3	16.8 ± 3.1	0.0854
Cu, PPM	5.8 ± 1.2	6.0 ± 0.7	7.2 ± 0.8	0.0503
Mn, PPM	66.0 ± 21.1	84.4 ± 24.3	75.2 ± 16.5	0.3631
Starch, %	0.4 ± 0.1^b^	0.7 ± 0.1^a^	0.6 ± 0.2^a^	0.0049
WSC^*e*^, %	4.0 ± 1.3	4.7 ± 0.7	4.4 ± 1.5	0.6642
ESC^*f*^, %	3.2 ± 1.4	3.2 ± 0.9	2.2 ± 1.5	0.3223
NSC^*g*^, %	4.4 ± 1.3	5.4 ± 0.8	5.0 ± 1.4	0.4275

Values with differing letters are significantly different at *P* < 0.05.

^*a*^Treatment: BB = big bluestem hay; IG = indiangrass hay; OG = orchardgrass hay.

^*b*^Crude protein.

^*c*^ Acid detergent fiber.

^*d*^Neutral detergent fiber.

^*e*^Water-soluble carbohydrates.

^*f*^Ethanol-soluble carbohydrates.

^*g*^Nonstructural carbohydrates.

#### Toxicological markers.

 Biomarkers for toxicity stayed within parameters deemed acceptable by our veterinarian based on accepted normal ranges and prior toxicological research on our research farm (Mercer et al., 2020), with one exception. For one horse, ALP, AST, bile acid, GGT, SDH, and total bilirubin were elevated past acceptable ranges on the final sampling of period 1. The horse was immediately removed from the study and was sampled frequently to monitor biomarkers. The horse did not exhibit clinical symptoms at any time. As no other horses were affected, and the horse’s biomarkers remained elevated after removal from the hay and turnout on cool-season mixed pastures, our veterinarian determined the elevated biomarkers most likely indicated either a response to an unknown insult or a recurrence of a past medical issue. The horse was on the BB treatment; no other horse had a similar response to consuming the BB hay. Consequently, we omitted that horse from toxicological analyses. In periods 2 and 3, an alternative horse was used.

Most biomarkers did not differ among treatments (Table 3). Of those that did, GGT increased the most in the IG treatment (*P* = 0.0166) and SDH decreased the most (*P* = 0.0377). Triglycerides were lower in BB and IG treatments than in the OG treatment (*P* = 0.0062).

**Table 3.** Biomarker change from baseline values in horses fed big bluestem, indiangrass, and orchardgrass hays

	Treatment^*a*^			
Biomarker	BB	IG	OG	*P-*value
Albumin, g/dL	0.0 ± 0.4	0.1 ± 0.2	−0.5 ± 0.5	0.0667
ALP^*b*^, U/L	−0.6 ± 23.8	48.0 ± 54.4	−8.0 ± 39.9	0.1061
AST^*c*^, U/L	−101.1 ± 42.8	−54.5 ± 26.2	−57.6 ± 34.7	0.3466
Bile acid, µmol/L	−0.3 ± 1.2	−0.4 ± 1.2	−2.5 ± 2.1	0.1245
Direct bilirubin, mg/dL	0.0 ± 0.1	−0.1 ± 0.1	0.0 ± 0.1	0.6948
GGT^*d*^, U/L	1.0 ± 3.1^ab^	5.7 ± 4.2^a^	0.0 ± 1.9^b^	0.0166
SDH^*e*^, U/L	−5.1 ± 2.5^ab^	−3.5 ± 6.6^a^	−12.9 ± 8.5^b^	0.0377
Total bilirubin, mg/dL	0.7 ± 0.4	0.7 ± 0.4	0.5 ± 0.3	0.7592
Triglycerides, mg/dL	−3.5 ± 10.2^b^	−3.0 ± 7.7^b^	16.0 ± 11.9^a^	0.0062

Treatment means with different letters are statistically different at *P* < 0.05. Data presented are least squares means ± SE.

^*a*^Treatment: BB = big bluestem hay; IG = indiangrass hay; OG = orchardgrass hay.

^*b*^Alkaline phosphatase.

^*c*^Aspartate aminotransferase.

^*d*^Gamma glutamyl transferase.

^*e*^Sorbitol dehydrogenase.

#### Intake.

 Voluntary DMI was greater for OG than either of the NWSG hays, both in terms of mass-consumed (*P* = 0.0042) or percent BW (*P* = 0.002) (Table 4). Horses lost weight on both NWSG species tested, but gained weight on OG (*P* = 0.0357). For all measures of intake or change in BW, there was a treatment effect, but no period effect or period × treatment interaction was detected.

**Table 4.** Mean dry matter intake and change in bodyweight by treatment

	Treatment^*a*^			
Variable	BB	IG	OG	*P-*value
DMI, kg/d	6.6 ± 0.8^b^	7.0 ± 0.9^b^	8.9 ± 1.8^a^	0.0042
DMI, % of BW	1.1 ± 0.1^b^	1.3 ± 0.1^b^	1.7 ± 0.3^a^	0.0020
Change in BW	−16.3 ± 19.0^ab^	−21.2 ± 26.2^b^	3.8 ± 25.6^a^	0.0357

Treatment means with different letters are statistically different at *P* < 0.05. Data presented are least squares means ± SE.

^*a*^Treatment: BB = big bluestem hay; IG = indiangrass hay; OG = orchardgrass hay.

#### Digestibility.

Apparent DM digestibility did not differ among treatments (Table 5). Crude protein was more digestible for OG than in BB or IG (*P* < 0.0001). Starch was more digestible in OG and IG than BB (*P* = 0.0096). The IG treatment had higher apparent digestibility of Ca (*P* = 0.0002), Mn (*P* = 0.0065), and Cu (*P* = 0.0033).

**Table 5.** Apparent digestibility of the three treatment hays fed

	Treatment^*a*^			
Digestibility, %	BB	IG	OG	*P-*value
Dry matter	38.8 ± 10.1	40.6 ± 8.4	43.2 ± 5.3	0.2636
CP^*b*^	18.3 ± 13.5^b^	30.1 ± 14.4^b^	52.4 ± 3.9^a^	<0.0001
ADF^*c*^	51.3 ± 9.2	46.7 ± 7.7	48.5 ± 5.6	0.0979
NDF^*d*^	47.6 ± 9.0	45.7 ± 8.0	46.5 ± 5.5	0.6415
Ca	−24.0 ± 26.1^b^	15.6 ± 17.4^a^	−13.7 ± 22.7^b^	0.0002
P	−55.4 ± 21.5^b^	−30.3 ± 13.1^a^	−47.6 ± 30.4^ab^	0.0076
Mg	−20.6 ± 21.7	−23.2 ± 22.9	−13.0 ± 9.6	0.5823
K	58.7 ± 12.7	57.7 ± 11.7	57.3 ± 9.0	0.8599
Fe	−213.2 ± 145.2	−144.6 ± 107.0	−89.3 ± 87.7	0.0561
Zn	−34.0 ± 27.9	−29.3 ± 20.6	−45.1 ± 28.3	0.3722
Cu	−14.6 ± 18.0^b^	13.3 ± 14.6^a^	−8.6 ± 10.0^b^	0.0033
Mn	−77.0 ± 50.8^b^	−18.8 ± 23.2^a^	−60.0 ± 22.5^b^	0.0065
Starch	−27.0 ± 86.6^b^	42.7 ± 19.8^a^	39.5 ± 17.9^a^	0.0096
WSC^*e*^	55.7 ± 17.9	66.5 ± 7.0	59.4 ± 5.5	0.1806
ESC^*f*^	66.1 ± 24.9	66.0 ± 19.1	81.3 ± 9.0	0.0588

Treatment means with different letters are statistically different at *P* < 0.05. Data presented are least squares means ± SE.

^*a*^Treatment: BB = big bluestem hay; IG = indiangrass hay; OG = orchardgrass hay.

^*b*^Crude protein.

^*c*^Acid detergent fiber.

^*d*^Neutral detergent fiber.

^*e*^Water-soluble carbohydrates.

^*f*^Ethanol-soluble carbohydrates.

### Experiment II

#### Forage characteristics.

 Available forage differed at the beginning of the grazing trial. BB and IG standing biomass did not differ (about 3000 kg/ha), but the GG treatment had much lower available forage (410 kg/ha) (*P* < 0.0001). Weedy species biomass also differed, with BB having the lowest weedy biomass at 130 kg/ha and GG having the most, at 506 kg/ha (*P* = 0.0002). At the end of the July grazing bout, available forage did not differ among treatments, ranging from 386 kg/ha in the GG plots to 565 kg/ha in the BB plots. Weedy biomass differed again, with GG having greater weedy biomass at 580 kg/ha than both the BB and IG plots, at 62 and 295 kg/ha, respectively (*P* = 0.0003).

At the beginning of the September grazing bout, IG pastures had the most available forage (1,450 kg/ha) while GG again had the least (250 kg/ha) (*P* = 0.0354). BB pasture yields were intermediate (620 kg/ha). The GG treatment again had the greater weedy species biomass, increasing to 780 kg/ha compared with 330 kg/ha and 170 kg/ha in the IG and BB treatments, respectively (*P* = 0.0002). After the September grazing bout, there were no differences in available forage among treatments.

Total seasonal forage availability differed among treatments, with the highest mean total available forage in the IG treatment, at 4,340 kg/ha, and the lowest in the GG at 650 kg/ha (Table 6) (*P* = 0.0140). Overall forage removed also differed between treatments, with the highest mean removal on IG plots at 3,880 kg/ha, and the lowest removal on GG, which had net negative removal over the course of the grazing trial, indicating an increase in biomass of about 260 kg/ha (*P* = 0.0070).

**Table 6.** Mean available and removed NWSG forage and weed biomass (kg/ha) of treatments in the grazing trial

		NWSG		Weeds	
	Treatment^*a*^	Available	Removed	Biomass	Removed
July	BB	2970	2400	130	70
	GG	400	20	900	310
	IG	2900	2460	510	210
September	BB	620	440	170	80
	GG	250	−280	780	240
	IG	1460	1410	330	180
Total	BB	3590	2890	300	140
	GG	650	−260	1680	550
	IG	4340	3880	830	390
*P*-value		0.014	0.007	0.0025	0.188

Removed forage was the difference between standing NWSG biomass at the start and finish of each grazing bout. A negative value indicates a gain rather than loss in biomass. *P* values reported are for repeated measures ANOVA comparing treatments across the course of the experiment.

^*a*^Treatment: BB = big bluestem; IG = indiangrass; OG = orchardgrass.

**Figure 1.**
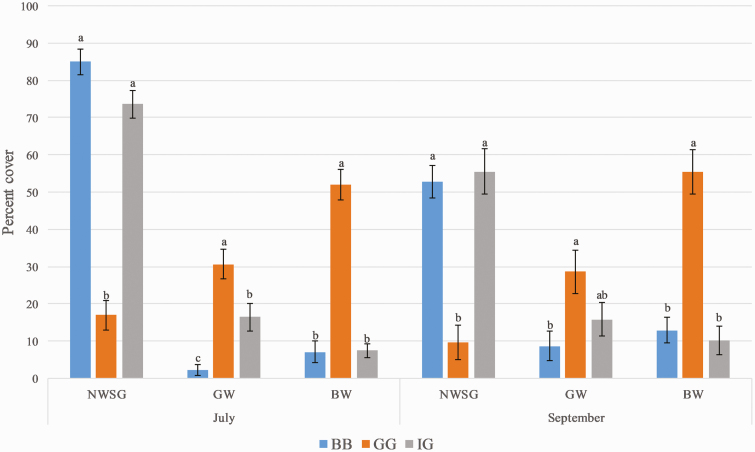
Percent cover of native warm-season grasses (NWSG), grassy weeds (GW), and broadleaf weeds (BW) in July and September at the start of each grazing bout for the big bluestem (BB), gamagrass (GG), and indiangrass (IG) plots. Means with different letters are significantly different (*P* < 0.05).

Percent cover of IG and BB did not differ in July or September at the start of each grazing bout (Figure 1). BW had higher percent cover in GG plots than IG or BB in July (*P* < 0.0001) and September (*P* < 0.0001). GW had higher percent cover in GG than in IG or BB in July (*P* < 0.0001), and were higher in GG than in BB in September (*P* = 0.0160).

After being grazed in July, over half the cover of BB and IG plots was comprised of trampled NWSG biomass, while no GG was trampled (*P* < 0.0001) (Table 7). After the September grazing bout, about a third of the cover in BB plots was trampled NWSG, and IG plots once again were comprised of over 50% trampled forage from the most recent grazing bout. Again, GG plots had no measurable trampling of native grass (*P* < 0.0001).

**Table 7.** Mean percent cover of trampled NWSG by treatment following each grazing bout

	Grazing bout	
Species^*a*^	July	September
BB	67.7 ± 32.0^a^	36.3 ± 34.1^a^
GG	0.0 ± 0.0^b^	0.0 ± 0.0^b^
IG	52.5 ± 34.4^a^	54.5 ± 38.7^a^
*P*-value	<0.0001	<0.0001

Means not connected by the same letter are significantly different at *P* < 0.05.

^*a*^Species: BB = big bluestem; IG = indiangrass; OG = orchardgrass.

Biomass samples of NWSG from each plot were subsampled and composited for nutritional analysis at the beginning of the first grazing trial. Due to the sparseness of GG, nutritional analysis could not be carried out on the harvested samples of two of the three plots. Consequently, only BB and IG were compared statistically. They did not differ in CP, ADF, NDF, DE, or NSC (Table 8).

**Table 8.** Forage nutritive value indices for big bluestem (BB), eastern gamagrass (GG), and indiangrass (IG) treatments in the grazing trial. No SD is reported for gamagrass because only one sample had sufficient biomass for chemical analysis

Species^*a*^	CP, %	ADF, %	NDF, %	DE, Mcal/kg	NSC (%)
BB	7.0 ± 0.3	45.5 ± 2.6	74.5 ± 0.5	1.5 ± 0.8	9.9 ± 1.3
GG	6.2	41.7	74.7	1.1	12.4
IG	6.0 ± 0.8	45.0 ± 0.9	74.7 ± 1.5	1.1 ± 0.6	8.3 ± 1.4
*P-*value	0.1419	0.7495	0.8117	0.4854	0.2090

Only BB and IG were compared statistically.

^*a*^Species: BB = big bluestem; IG = indiangrass; OG = orchardgrass.

#### Animal weight.

**Figure 2.**
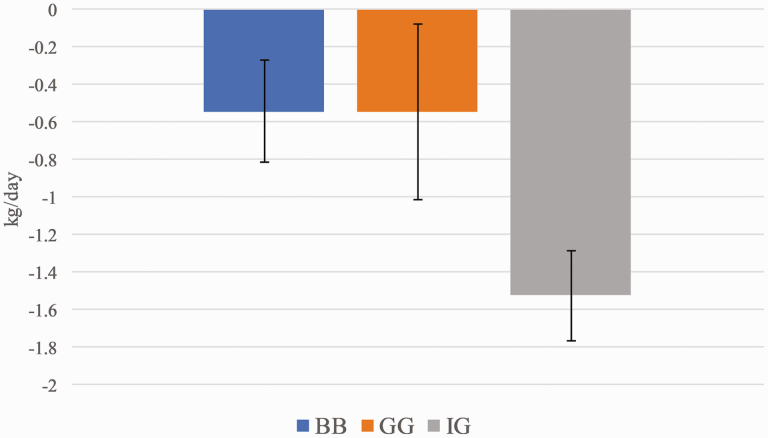
Average changes in equine bodyweight on the big bluestem (BB), gamagrass (GG), and indiangrass (IG) treatments during the grazing experiment expressed as kilograms per day with standard error.

Horses lost weight on all treatments during the grazing bouts (Figure 2). Weight changes in horses did not differ among treatments; however, there was a trend for horses on the IG treatment to lose more weight than those on the BB or GG treatments, at 1.5 kg/day lost on IG plots and about 0.5 kg/day in the other two treatments (*P* = 0.0977).

## DISCUSSION



#### Experiment I.

 The objectives of experiment I were to determine if BB and IG hays induce a toxic response in horses after 2 wk of feeding and to evaluate the potential use of these species as a hay source for equines. Biomarkers of toxicity did not exceed acceptable limits after 2 wk. Voluntary intake of these forages was lower than orchardgrass intake in our study and lower than reported values for other species. However, this may be ideal for horse owners struggling to find optimal forages for horses prone to obesity and laminitis. Apparent digestibility of some nutrients was low or negative for the NWSG hays and orchardgrass on our study, indicating a need to supplement these forages with a ration balancer to ensure adequate intake of vitamins, minerals, and protein.

### Toxicological Response

The two NWSG hays tested did not cause biomarkers of toxicity to exceed levels deemed safe by the veterinarian monitoring horses on this study based on prior toxicological research conducted with Thoroughbred geldings on this site (Mercer et al., 2020). As discussed above, one horse on the BB treatment had elevated biomarkers, but the lack of any similar response in the other horses fed the hay as well as the continued elevated biomarkers when that horse consumed cool-season pasture during a washout and monitoring period suggest a cause other than diet. Additionally, we can find no case reports in the literature of BB hay causing a toxic response in horses.

Among biomarkers measured, only GGT increased in an NWSG hay (IG) relative to the OG treatment. GGT is an indicator of hepatic function, and changes were slight. To determine if this response indicates the potential for hepatic insult caused by IG hay, a longer feeding trial may be necessary for future research. Additionally, our results cannot rule out the possibility of cystitis-ataxia for horses consuming a diet of IG long-term, as cystitis-ataxia does not affect the liver and is diagnosed clinically rather than by blood samples (Adams et al, 1969). None of our horses exhibited clinical signs of cystitis-ataxia after 2 wk of consuming IG; however, longer feeding trials should be conducted to determine if IG poses a risk for horses in that regard.

The declines in AST and SDH for all treatments during the feeding trial were likely a result of the horses switching from a species-rich pasture during the washout periods to monospecific hay. On pasture, horses had exposure to greater varieties of forbs and grasses, some of which can cause hepatic insult. When fed a diet exclusively consisting of one grass species, this exposure is eliminated. A similar decline was measured in a study of acetaminophen pharmacokinetics in Thoroughbred geldings at the same facility when horses were removed from pasture and fed only hay (Mercer et al., 2020).

Triglycerides increased in the OG treatment but decreased slightly in the NWSG treatments, which can be explained by the loss of BW on the NWSG treatments and increase in BW on OG. Moderate weight loss in humans results in lower serum triglyceride levels (Andersen et al., 1995). Similarly, Suagee et al. (2013) reported a positive relationship between equine body condition and plasma triglycerides, as well as insulin concentration and plasma triglycerides. These results align with our measure of limited increases in triglycerides for horses experiencing a minor increase in BW.

### Voluntary Dry Matter Intake and Nutritive Values

BB matures earlier than IG, and as the fields for both treatments were cut the same week, BB hay had reached a more mature stage with a higher proportion of reproductive tillers, while IG was still vegetative. Orchardgrass hay was also in a vegetative state when cut in late spring. As hay increases in maturity, nutritional quality decreases and voluntary intake by horses declines (Staniar et al., 2010). The NWSG hays in our study were cut later than is optimal, as prolonged rain-delayed opportunities for cutting and curing at the site of harvest. Nutritive values from a mixed hayfield of IG and BB from 2010 to 2012 in Tennessee (Keyser et al., 2012) averaged 66.8% NDF, 40.2% ADF, and 9.3% CP—substantially lower fiber concentrations and moderately higher CP than the NWSG hay in our study.

Voluntary DMI of the NWSG hays was lower than values reported for mature warm-season grasses in past studies, such as 2.1% for “Coastal” bermudagrass (*Cynodon dactylon*) or 2.3% for Caucasian bluestem (*Bothriochloa bladhii*) (Crozier et al., 1997; LaCasha et al., 1999). Voluntary DMI for horses consuming OG was within the normal ranges of 1.5–3.1% BW described in the National Research Council’s guidelines for equine nutrition, with IG falling slightly below the normal range at 1.3% and BB well below at 1.1% (National Research Council, 2007). However, for obese horses needing reduced digestible energy intake, Virginia Cooperative Extension recommends reducing hay intake to 1–1.5% of the target BW while maintaining constant forage availability to minimize risk of gastric ulcers (Porr and Crandell, 2008).

Differences in NDF paralleled differences in intake among treatments, with OG having the lowest NDF and highest intake, and the NWSG having high NDF values and lower intake by horses. This aligns with past research demonstrating the value of NDF as a predictor of intake, with lower NDF levels predicting higher voluntary intake (LaCasha et al., 1999).

### Digestibility

Dry matter digestibility (DMD) measured in our study ranged from 38.8% to 43.2%, similar to values reported for lower-quality hay in previous research, such as 43% in coastal bermudagrass (Aiken et al., 1989), 44% in Caucasian bluestem (*Bothriochloa bladhii*) (Crozier et al., 1997), 38.5% in reed canarygrass (*Phalaris arundinacea*), and 42.1% in crested wheatgrass (*Agropyron cristatum*) (Cymbaluk, 1990). Apparent DMD of the hay treatments in our study was lower than those reported for another warm-season grass, teff, at three different stages of maturity, which ranged from 51.5% to 60.6% when fed to horses (Staniar et al., 2010). The DMD of NWSG hays in our study were also lower than those reported for alfalfa, popular hay for horses, which ranges from 58% to 64% (Crozier et al., 1997; Sturgeon et al., 2000). The NWSG hays, while not highly digestible, are adequate as roughage for equines susceptible to metabolic disorders.

### Integrating NWSG Hays into the Equine Diet

Based on published nutrient requirements (National Research Council, 2007), the NWSG hays fed in our study do not meet DE requirements for a mature horse at maintenance at the intake rates we observed. However, this may be advantageous for horses prone to obesity and laminitis, as reducing dietary energy and NSC in the diet of horses prone to obesity and laminitis is recommended (Geor, 2009). Additionally, the low concentration of NSC in the NWSG hays is ideal for horses susceptible to carbohydrate-induced laminitis, as they were about half the recommended maximum range of 10–12% (Geor, 2009). The loss of BW observed on NWSG hays in our study was acceptable, especially given that the horses in this study were hard-keeping off-track Thoroughbreds and the trial took place in midwinter, when horses require more energy to maintain thermal homeostasis. As such, the energy levels in these hays may be ideal for horses in need of a “diet” hay, resulting in optimal weight loss rather than either extreme loss or weight gain.

However, trace minerals and vitamins may be deficient in a diet of only BB or IG hay based on nutrient values in our hay and observed intake rates, and the ratio of Ca:P was approximately 1:1 for the BB and 3:2 for IG rather than the optimal 2:1 recommended for horses (National Research Council, 2007). Additionally, CP was low in the NWSG hays, indicating a necessity for protein supplementation if fed long term. As such, it would be advisable for equine managers to supplement the horse’s diet with a ration balancer formulated to compensate for these deficiencies. This is recommended for all horses on pasture in Virginia, however, and as such should not pose an additional challenge in nutritional management (Porr and Greiwe-Crandell, 2009).

Impaction colic should also be considered when weighing the risks and benefits of NWSG hay. While impaction colic did not occur in the horses on this study, high fiber content in hay is a contributing factor to potential impactions. Impactions have been reported on other warm-season grasses high in fiber, such as bermudagrass and teff (Little and Blikslager, 2002; Staniar et al., 2010).

#### Experiment II.

This study evaluated three NWSG species to determine their potential suitability as pasture grasses for horses in Virginia. Our results suggest both IG and BB could be used as pasture grasses for horses in summertime if grazed at a suitable stocking rate to ensure the long-term survival of the stand. Gamagrass plots were not sufficiently established for effective evaluation of response to grazing.

### Forage Productivity

Forage biomass and regrowth in IG and BB were adequate to sustain each of the horses in this study on 0.1 ha for about 3 wk, with total available forage of 4,344 kg/ha for IG and 3,587 kg/ha for BB. Had the plots experienced another year of establishment prior to the grazing trial, their forage production would have been higher, as NWSG do not reach full productivity until their second or third year of establishment (Harper et al., 2011). Mature, fertilized stands of BB and IG produced 6,290 and 5,590 kg of dry matter/ha, respectively, in yield trials in Iowa (Hall et al., 1982). Forage yields of these two species in Tennessee typically range from 5,600 to 9,000 kg/ha (Harper et al., 2011). As these plots were unfertilized monocultures grazed a year after establishment, the lower yields relative to other studies are to be expected.

Differences in the seasonal timing of growth between species in our study may have impacted the quantity of forage regrowth between grazing bouts. The IG plots had higher regrowth than BB (940 kg/ha vs. 50 kg/ha, respectively). The BB plots had produced reproductive tillers prior to the onset of the grazing trial, and may have used more of their carbohydrate reserves producing reproductive tillers than IG as a result before being grazed, limiting available reserves for regrowth. IG plots did not produce reproductive tillers until after the first grazing trial. BB generally reaches maturity earlier in the season than IG (Keyser et al., 2012). Mixing these two grasses in a pasture could provide more uniform seasonal forage distribution than managing as monocultures; however, binary mixtures of these species may not appreciably increase overall yields compared to their monocultures, as a biofuel trial in the northern Great Plains found (Hong et al., 2013).

### Animal Weight

The trend for greater weight loss in horses on IG plots was unlikely to be a result of differences in forage nutritive value, as DE and CP were similar between forage species. Higher forage intake on BB compared to IG may account for the trend; however, DMI was not measured during the grazing trial. While no studies have compared the palatability of these NWSG species for horses, Dwyer and Sims (1964) conducted a palatability trial of common prairie species with steers and found BB to be among the most palatable forages, with IG intermediate among the 18 species tested. Though biomass of GG was lower than either of the other two species, the abundance of palatable weeds in those plots such as white clover and crabgrass could in part explain the lower rate of weight loss by horses on those plots relative to IG, as many weedy species common to Virginia pastures have high nutritive value (Abaye et al., 2009).

### Trampling

Our study did not directly measure DMI from grazing, making it impossible to determine how much of the forage removed during each grazing bout was through ingestion rather than trampling. However, both BB and IG plots had more than 50% trampled NWSG cover after the first grazing bout, and about 35–55% trampled NWSG cover after the second bout, suggesting the loss of forage to trampling was considerable. As the grazing trial did not begin until July, both BB and IG were tall and more susceptible to lodging than they would have been earlier in the growing season.

Trampling damage is an important aspect of equine impacts on pasture (Bott et al., 2013). While trampling simulations have been conducted to measure traffic tolerance in turf species for horses (Jaqueth, 2019), or to determine the effect of trampling by feral horses in coastal ecosystems (Turner, 1987), we found no studies quantifying pasture forage losses for actual, rather than simulated, trampling by equines. A study measuring cattle trampling impacts on pasture found that a single trampling event reduced daily growth from 18 to 11kg/ha/day on a pasture in New Zealand, and the effect persisted for more than 7 wk (Pande et al., 2000). Equines can move faster than cattle, and their different hoof morphology may lend itself to greater sward damage. Reductions in sward yield and vigor by equine trampling may be more pronounced. Future studies of equine grazing dynamics on NWSG swards would benefit from incorporating sward height measurements, as well as determinations of equine DMI to provide more definitive assessments of the relative importance of herbivory and trampling on reductions of available forage.

## CONCLUSIONS

This study evaluated horse responses in a hay feeding trial and grazing experiment using three NWSG species in Virginia. BB and IG hay elicited no toxic effects in horses for the biomarkers we measured. Voluntary intake on NWSG hays was lower than on more common cool-season grass hays, which may be ideal for horses susceptible to metabolic disorders such as obesity and laminitis. Horse owners with animals prone to these disorders should consider integrating these two species into their equine ration in combination with a ration balancer or mineral block.

In pasture, BB and IG are productive species that can provide useful alternative summer forages for equine grazing systems. Gamagrass establishment was poor in our grazing experiment but GG increased in productivity over the course of the grazing trial. For horse owners or land managers interested in forage options other than high-carbohydrate cool-season pastures, IG and BB provide an opportunity to optimize equine summer grazing for horses vulnerable to obesity and pasture-associated laminitis. If grazing NWSGs with horses, stocking rate determinations should take the likelihood of high forage losses to trampling damage into account.
